# Associations Between Maternal Stressful Life Events and Perceived Distress during Pregnancy and Child Mental Health at Age 4

**DOI:** 10.1007/s10802-022-00911-7

**Published:** 2022-03-08

**Authors:** Kristen L. Rudd, Sylvia S. Cheng, Alana Cordeiro, Michael Coccia, Catherine J. Karr, Kaja Z. LeWinn, W. Alex Mason, Leonardo Trasande, Ruby H. N. Nguyen, Sheela Sathyanarayana, Shanna H. Swan, Emily S. Barrett, Nicole R. Bush

**Affiliations:** 1 Department of Psychiatry and Behavioral Sciences, University of California San Francisco, San Francisco, CA USA; 2 Department of Pediatrics, University of Washington, Seattle, WA USA; 3 Weill Institute of Neuroscience, University of California San Francisco, San Francisco, CA USA; 4 Department of Preventative Medicine, University of Tennessee Health Science Center, Memphis, TN USA; 5 Department of Pediatrics, New York University Langone Medical Center, New Yok, NY USA; 6 Departments of Population Health and Environmental Medicine, New York University, New York, NY USA; 7 Department of Epidemiology and Community Health, University of Minnesota, Minneapolis, MN USA; 8 Department of Environmental Medicine & Public Health, Icahn School of Medicine at Mount Sinai, New York, NY USA; 9 Department of Biostatistics and Epidemiology, Occupational Health Sciences Institute, Rutgers School of Public Health, Piscataway, NJ USA; 10 Department of Pediatrics, University of California San Francisco, CA San Francisco, USA

**Keywords:** Stressful life events, Perceived distress, Prenatal programming, Internalizing, Externalizing, Adaptive skills

## Abstract

Accumulating evidence suggests that maternal exposure to objectively stressful events and subjective distress during pregnancy may have intergenerational impacts on children’s mental health, yet evidence is limited. In a multisite longitudinal cohort (N = 454), we used multi-variable linear regression models to evaluate the predictive value of exposure to stressful events and perceived distress in pregnancy for children’s internalizing problems, externalizing problems, and adaptive skills at age 4. We also explored two- and three-way interactions between stressful events, distress, and child sex. Both objective and subjective maternal stress independently predicted children’s behavior, with more stressful events and higher distress predicting more internalizing and externalizing problems and worse adaptability; stress types did not significantly interact. There was some evidence that more stressful events predicted higher externalizing behaviors only for girls. Three-way interactions were not significant. The current findings highlight the importance of considering the type of stress measurement being used (e.g., counts of objective event exposure or subjective perceptions), suggest prenatal stress effects may be transdiagnostic, and meet calls for rigor and reproducibility by confirming these independent main effects in a relatively large group of families across multiple U.S. regions. Results point to adversity prevention having a two-generation impact and that pre- and postnatal family-focused intervention targets may help curb the rising rates of children’s mental health problems.

**Supplementary Information:**

The online version contains supplementary material available at 10.1007/s10802-022-00911-7.

Over the past decade, advances in research have uncovered the importance of prenatal environments for children’s postnatal mental health outcomes (Hartman & Belsky, 2018; Monk et al., 2019). The Developmental Origins of Health and Disease framework, and supporting empirical data, suggest that in addition to nutrition and environmental toxicants, maternal experiences of stress during pregnancy influence the development and mental health of their children (Graignic-Philippe et al., 2014). These experiences of stress are understood to alter endocrine and immune processes that play a crucial role in early fetal neurodevelopmental programming, operating via the placental connection between mother and fetus (Monk et al., 2019). Exposure to stressful experiences can include a diverse range of occurrences, such as relationships marked by extreme conflict or abuse or severe illness or death of a relative. However, individuals often vary in their perception of distress related to exposures that are widely considered “objectively” stressful. Thus, the range of existing stress measures used in prenatal programming research may be capturing different aspects of “stress” (Epel et al., 2018). As such, it is important to consider both objective and subjective measures of stress when estimating the potential intergenerational impact of women’s pregnancy experiences on child mental health.

Studies utilizing subjective measures of distress such as daily hassles, pregnancy related anxiety, or perceived stress have shown that maternal reports of high distress during pregnancy are positively associated with children’s psychopathological outcomes (e.g., total behavior problems, externalizing internalizing; Hentges et al., 2019; Lazinski et al., 2008; Racine et al., 2018). More objective measures of stress, such as whether or not women have experienced stressful events (e.g., domestic violence, death of a relative) follow similar trends, with higher counts of stressful events relating to more child externalizing problems (Robinson et al., 2011). However, few studies have simultaneously explored exposure to objectively stressful events and subjective distress measures during pregnancy in the same model to evaluate whether there are additive (or interactive) effects of variations in perceptions of distress in response to experiencing stressful events. As a notable exception, a recent study harnessing measures of both perceived stress during pregnancy and retrospective report of exposure to pregnancy stressful events (reported at 6-months postpartum) found that women’s exposure to stressful events was associated with infant temperament and biological stress-reactivity outcomes, but only at moderate to high levels of perceived stress (Bush et al., 2017). Including evaluations of multiple types of stress measures in longitudinal dyadic research can advance the field’s current understanding of when, how, and what type of experiences of stress have effects on children’s development of psychopathology across generations.

Further, existing studies of maternal pregnancy stress and child mental health typically take a deficit-focused lens. Although understanding the effect of stress on child dysfunction is important, even less is known about whether similar intergenerational transmission pathways operate to impact more positive mental health outcomes, such as children’s ability to adapt to changes in their environment in a manner that leads to better functioning and positive health (not just the lack of disease). By harnessing multiple types of stress as well as multiple indicators of childhood health and disorder, research can advance theories of how specific disorders may develop and whether different types of experiences (e.g., objective experiences versus subjective perceptions) may contribute to different aspects of children’s socioemotional health. Specifically, research focused on identifying presyndromal effects in community samples may be particularly important for prevention efforts aimed at curbing the rising rates of childhood psychopathology by targeting those on trajectories of early risk.

Some research also suggests that the biological sex of the developing fetus may modify the effects of maternal prenatal stress on child mental health via sex-specific differences in hormonal mechanisms (Glover & Hill, 2012; Sutherland & Brunwasser, 2018). However, findings are mixed as to whether males or females are more susceptible to prenatal maternal stress (Van den Bergh & Marcoen, 2004; Van den Bergh et al., 2008), while others find no significant differences by child sex (Hentges et al., 2019). Collectively, inconsistency in the emerging literature suggests more longitudinal research is needed in larger samples of children and with a range of positive and negative health outcomes to fully understand potential sex-specificity in prenatal programming of children’s mental health and well-being.

To address these gaps, the current study used longitudinal data from a multi-site U.S. study to evaluate the simultaneous independent associations between maternal report of exposure to stressful events and perceived distress during pregnancy for children’s internalizing problems, externalizing problems, and adaptive skills at age 4, while adjusting for important pre- and postnatal confounders. We hypothesized that there would be additive effects of both exposure to events and distress such that children of women who experienced more types of stressful events and/or higher perceived distress during pregnancy would have higher reported internalizing and externalizing problems and lower adaptive skills at age 4. Further, given early evidence that there may be interactive effects between objective events and subjective perceptions, as well as modifying effects of the sex of the developing fetus, we explored both two- and three-way interactions. In line with previous findings, we hypothesized that there would be a significant interaction between types of stress such that women’s exposure to more pregnancy stressful events would be associated with higher internalizing and externalizing problems and lower adaptive skills in children only when women also reported moderate-to-high levels of distress. Given the inconsistencies in the limited literature, we had no apriori directional hypotheses regarding sex differences.

## Methods

### Participants and Procedures

The current study leverages an ongoing prospective cohort study designed to examine prenatal phthalate exposure in relation to child development. Full information on participant enrollment and exclusion has been described in previous publications (Barrett et al., 2014). Women were recruited during their first trimester of pregnancy from clinics at four U.S. academic medical centers (University of California, San Francisco; University of Washington, Seattle; University of Minnesota; and University of Rochester) between 2010 to 2012. Eligibility criteria for the women included being less than 13 weeks pregnant, age 18 or older, having a singleton pregnancy, not having a medically threatened pregnancy, and planning to deliver in one of the study hospitals. Throughout the longitudinal study, women completed questionnaires in each trimester of pregnancy (N = 801 completed at least one pregnancy assessment), an evaluation at birth (N = 739 live births), a detailed follow-up assessment when the children were 4-years-old (*M*_*age*_ = 4.09 years, *SD* = 0.30; N = 542), and another when the children 6-years-old in order to gather retrospective reports of past exposure to stressful live events. Mother–child dyads were included in the current study if they had complete child mental health data at the age 4 assessment, yielding a final sample of 454 dyads, which is well powered to appropriately evaluate the hypothesized relations. Participants included in current study were 73% White, 9% Black/African American, 6% Asian American, 6% Multiracial, 11% Other. The majority of women were married or in committed relationships (84%) and had a bachelor’s degree or higher (82%); family median income was $90,300 at the age 4 assessment. Institutional Review Boards at all participating study sites (i.e., University of California, San Francisco; University of Washington, Seattle; University of Minnesota; and University of Rochester) approved the study prior to the start of study activities and all subjects signed informed consent.

### Measures

#### Maternal Pregnancy Stressful Life Events

Exposure to objectively Stressful Life Events during pregnancy (PSLE) was assessed via women’s reports using a list of 14 events from the Center for Disease Control and Prevention PRAMS survey (Whitehead et al., 2003) assessed retrospectively at the postnatal age 6 visit. Participants were asked to respond yes or no to statements about experiences with illness, death, relationship problems, housing difficulties, legal issues, and financial problems during pregnancy. Affirmative responses were summed. Recent evaluations of retrospective report of adverse events found moderate agreement between retrospective and prospective measures (Ramos et al., 2020; Reuben et al., 2016), supporting the validity of this measurement timing.

#### Maternal Pregnancy Perceived Distress

Subjective distress during pregnancy was assessed via women’s reports on their perceived distress during their second trimester of pregnancy using an adapted version of the Goldenberg abbreviated scale (Goldenberg et al., 1997). In addition to removing one item from the original scale, this adapted version also changed the wording of two items. Specifically, the item “I feel pleasant” became “I feel content” and the item “I feel content(satisfied)” became “I feel satisfied”. This 27-item measure assesses women’s general distress since their last assessment (mean = 22 weeks between assessments) across domains such as depression, anxiety, self-efficacy and demonstrates acceptable internal consistency in the present sample (Cronbach’s alpha = 0.937). Items included questions such as “I feel content”, “I have crying spells”, “I take a positive attitude towards myself”, and “I am easily bothered by things that didn't used to bother me”. Scores range from Never (*1*), Rarely (*2*), Sometimes (*3*), Often (*4*), and Almost Always (*5*). This study used the scale total average score, such that a higher score represents greater subjective distress (i.e., lower subjective well-being) during pregnancy, ranging from a lowest possible score of 1 to a highest possible score of 5.

#### Child Behavior

Mothers reported on their children’s behavior and self-perception at age 4 using the Behavior Assessment System for Children, Second Edition (BASC–2) (Reynolds & Kamphaus, 2004). The BASC-2 is a standardized measure of caregivers’ ratings of children’s behaviors, self-esteem, and adjustment abilities in the home and community. Responses range from not true of my child (*0*), to somewhat/sometimes true of my child (*1*), and almost always true of my child (*2*). The BASC-2 provided three composite summary scores characterizing symptoms of Internalizing Problems (40 items), Externalizing Problems (30 items), and Adaptive Skills (44 items). The Internalizing Problems summary score is comprised of anxiety, depression, and somatization subscale scores. The Externalizing Problems summary score is comprised of hyperactivity and aggression subscale scores. Finally, the Adaptive Skills summary score is comprised of activities of daily living, adaptability, functional communication, and social skills subscale scores. Raw scores for each subscale are scaled with respect to child age and sex, and then converted to t-scores for all analyses. Higher scores indicate more problematic behaviors for Internalizing and Externalizing Problems, but higher positive functioning for Adaptive Skills scores.

#### Covariates

We included several covariates that have been shown to relate to either maternal stress or children’s mental health outcomes. Women reported on their pre-pregnancy BMI (weight kg/height m^2^), as maternal obesity has been associated with fetal development and subsequent neurodevelopmental disorders in offspring (Reynolds et al., 2013). Gestational age at delivery as well as infant birthweight and sex were obtained via labor and delivery medical records. Maternal cigarette smoking and substance use during pregnancy, number of previous live births (parity), total household income, and household size were self-reported by women at their first trimester pregnancy visit. At the age 4 visit, mothers reported on their own highest level of education, perceptions of stress in the previous month using Cohen’s Perceived Stress Scale (PSS) (Cohen et al., 1983), and their depressive symptoms in the past 2 months using the Patient Health Questionnaire (PHQ-9; Kroenke et al., 2001) scale. Study site location was dummy coded with Rochester as the reference group to adjust for location.

### Analytic Plan

All analyses were conducted in R (version 4.0.2, R Core Team, 2021). Data were examined for non-normality to render parametric statistics valid following standard procedures (Afifi et al., 2007). Descriptive statistics were calculated for demographic characteristics of the sample. Bivariate correlations were used to examine associations among all study variables.

Multivariable regression analysis was used to evaluate independent and interactive relations between prenatal stress measures and children’s outcomes. Internalizing, Externalizing, and Adaptive Skills outcomes were assessed in separate models. Models were constructed in steps to enhance comparability to extant literature and evaluate the additive value of each set of additional variables. First, to address our main hypothesis, the predictor variables (i.e., PSLE and perceived distress) and all covariates listed above were included in the model (model 1). Second, we created interaction terms by multiplying each centered stress variable either by the other stress measure or by sex (e.g., PSLE x distress; PSLE x sex, or distress x sex) and entered the interaction terms into the model simultaneously (model 2). Finally, a 3-way interaction term, created by multiplying child sex by both stress measures, was entered into the model (model 3).

## Results

**Fig. 1:**
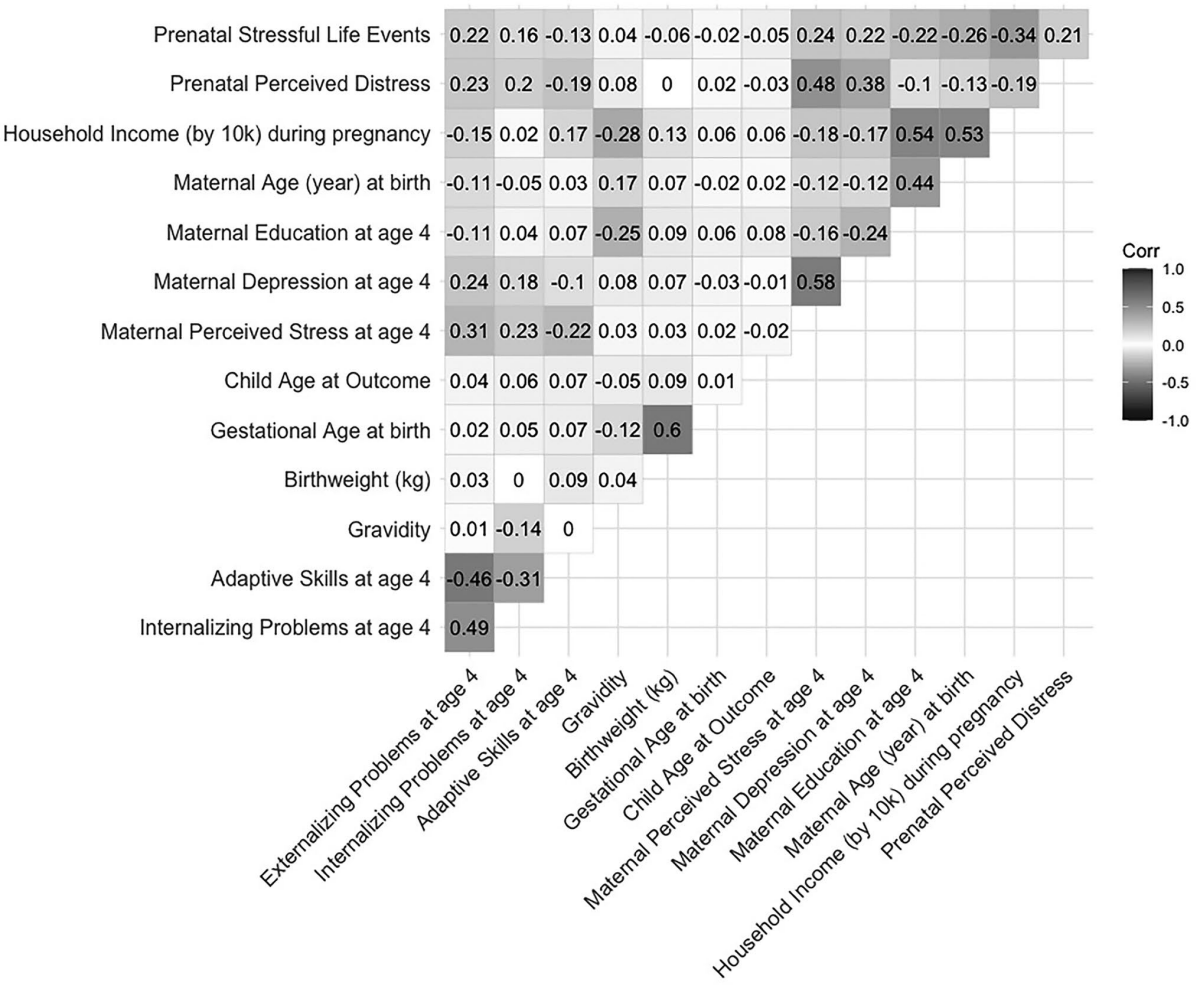
Bivariate relations between pregnancy stress measures, child mental health outcomes

The sample of participants included in the current analyses (i.e., those with child mental health data at age 4) did not significantly differ from the full pregnancy sample on any variables including key demographics, PSLE, and perceived distress (all *p* > 0.05). Table 1 presents demographic information on the current sample. Overall, there was a similar number of participants across the four study sites, however, independent samples t-tests revealed that the Rochester site had slightly higher reports of stressful life events, lower educational attainment, and lower income than other sites (all *p* < 0.05). Study sites did not statistically differ on any other measures. The number of PSLE women reported ranged from 0 to 8, and perceived distress scores ranged from 2.3 to 5.0. Of the total analytic sample, 13% of children were classified as “at-risk” for externalizing problems, and 3% reached levels of clinically significant problems. Similarly, 13% were classified as “at-risk” for internalizing problems, and 3% reached clinically significant level of problems. Adaptive skills had 15% of children classified as “at-risk”, and 3% who had clinically low adaptive skills. There was minimal comorbidity within this sample, with 5% of children being “at-risk” for both externalizing and internalizing problems and 1% meeting criteria for clinically significant levels of problems for both externalizing and internalizing. Although slightly lower than national averages, the prevalence of clinical level of problems in our study was similar to other studies of community samples at this young age (Basten et al., 2016; Eleni & Giotsa, 2018). Figure 1 displays the bivariate associations between study variables, including all covariates, PSLE, distress, as well as child behavioral problems and adaptive functioning at age 4.

**Table 1:** Descriptive information for the analytic sample of mothers and children

		**Total Sample** (N = 454)			
**Categorical Variables**		**N**		**%**	
Child Sex (female)		230		51%	
Study Site Location					
	San Francisco, California (UCSF)	124		27%	
	Seattle, Washington (UW)	91		20%	
	Rochester, New York (URMC)	116		26%	
	Minneapolis, Minnesota (UMN)	123		27%	
Marital Status					
	Married or living as married	384		84%	
	Single	45		10%	
	Separated, divorced, or widowed	25		6%	
Maternal Education at age 4					
	Less than college degree	80		18%	
	College degree or higher	374		82%	
**Continuous Variables**		**M**	**SD**		**Range**
Maternal Depression at age 4		0.33	0.38		0 – 2.55
Maternal Perceived Stress at age 4		1.41	0.58		.10 – 3.10
Child Age at outcome (year)		4.08	0.29		3.0 – 5.0
Gestational Age at birth (week)		39.30	1.81		25.00 – 42.43
Birthweight (kg)		3.36	0.56		0.55 – 5.15
Maternal Age (year) at birth		31.70	5.26		18.25 – 44.96
Gravidity		2.23	1.36		1 – 6
Household Income (by 10 k) during pregnancy		66.10	42.10		2.37 – 156.35
Pregnancy Stressful Life Events (count of types)		1.24	1.50		0 – 8
Pregnancy Perceived Distress		4.11	0.49		2.33 – 5.00
Child Behavioral Problems: Externalizing at age 4		49.50	8.03		33 – 78
Child Behavioral Problems: Internalizing at age 4		48.70	8.93		30 – 82
Child Behavioral Adaptive Skills at age 4		50.70	8.49		20 – 72

**Fig. 2:**
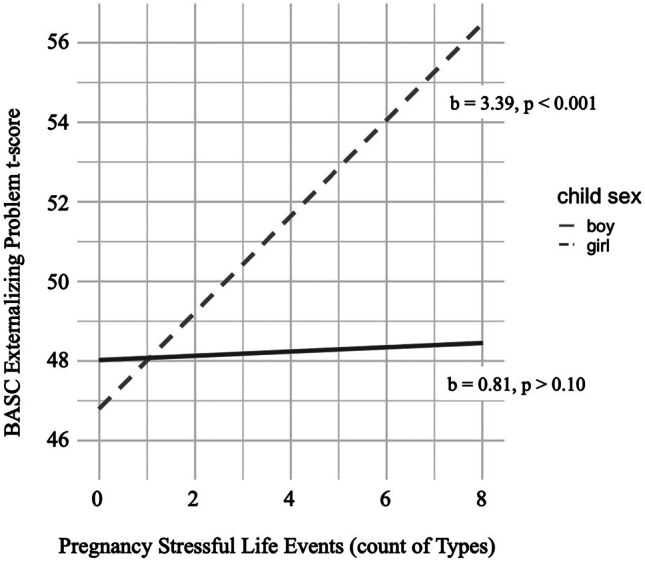
Two-way interaction between pregnancy stressful life events and child sex

Table 2 presents regression results from the main effect analyses. In models accounting for pre- and post-natal covariates, both stress measures independently predicted children’s mental health outcomes at age 4. Specifically, there was a significant main effect of PSLE such that women’s reports of exposure to more types of stressful life events during pregnancy was associated with higher levels of children’s Internalizing and Externalizing Problems (see Table 2). There was also a main effect of distress such that greater perceived distress during pregnancy was associated with higher levels of Internalizing Problems and lower levels of Adaptive Skills in children. Next, examining the two-way interaction among stressor types, PSLE and distress did not significantly interact to predict Internalizing (*B* = 0.64, *p* > 0.05), Externalizing (*B* = 0.10, *p* > 0.05), or Adaptive Skills (*B* = -0.27, *p* > 0.05); see Supplementary Table 1. Examining the two-way interactions between child sex and the stress measures, only one of six interactions tested was significant; see Supplementary Table 1. Specifically, exposure to PSLE interacted with child sex (*B* = 1.14, *p* <0.05) such that, for girls (simple slopes; *b* = 3.39, *p* < 0.001), maternal exposure to a greater number of types of stressful events was associated with higher levels of Externalizing Problems (see Fig. 2), whereas this this association was null for boys (simple slopes; *b* = 0.81, *p* > 0.10). Finally, the test for a possible three-way interaction among PSLE, distress, and child sex was not significant (*B* = -1.10, *p* > 0.05; results not shown in tables).

**Table 2:** Regression model results for tests of the main effect associations between PSLE and perceived distress on children’s age 4 outcomes

	**Externalizing**			**Internalizing**			**Adaptive Skills**	
Effect	B		SE	B		SE	B	SE
**Model 1**								
** Covariates**								
Child Sex (1 = female)	0.11	0.72		1.55	0.82		-0.43	0.79
Site = UMN	0.75	1.18		2.36	1.34		0.50	1.29
Site = UCSF	0.96	1.26		2.22	1.43		0.70	1.38
Site = UW	0.79	1.25		2.42	1.42		-0.47	1.37
Marital Status (1 = Married/living as married)	0.91	1.64		1.07	1.86		-1.71	1.79
Gestational Age at birth (weeks)	-0.10	0.26		0.18	0.29		0.27	0.28
Gravidity	-0.32	0.31		-0.86*	0.36		0.68*	0.34
Birthweight	0.67	0.84		-0.48	0.95		0.36	0.92
Smoking during pregnancy (1 = yes)	5.93**	1.90		3.76	2.15		-1.42	2.07
Substance use during pregnancy (1 = yes)	2.09	1.25		-0.14	1.45		0.45	1.37
Mother’s Education at age 4	0.22	1.30		1.21	1.48		0.22	1.42
Family Income during pregnancy (in $10,000)	-0.01	0.01		0.01*	0.01		0.04*	0.01
Child Age at outcome (years)	1.09	1.28		1.61	1.45		1.59	1.40
Maternal Depression at age 4	1.12	1.18		1.65	1.34		1.60	1.29
Maternal Perceived Stress at age 4	2.78***	0.808		1.97*	0.91		-2.69**	0.88
** Main Effects**								
Pregnancy Stressful Life Events (count of types)	0.57*	0.26		0.68*	0.30		-0.18	0.29
Pregnancy Perceived Distress	1.32	0.84		1.98*	0.95		-2.14*	0.92
	***R*** ^***2***^ ** = ** ***0.164***			***R*** ^***2***^ ** = ** ***0.132***			***R*** ^***2***^ ** = ** ***0.094***	

*Note*: Site comparisons were made with the Rochester, NY location as the reference group; PSLE = Pregnancy Stressful Life Events

^*^ *p* < .05, **p < .01, ﻿*** p < 0.001. Results from regressions testing 2- interactions can be found in Supplemental Table 1

We also conducted follow-up sensitivity analyses to examine whether pregnancy stressors independently predicted each child behavior outcome, while also including the other outcomes as predictors to account for shared variance among adaptive skills, internalizing problems, and externalizing problems. Pregnancy distress remained a statistically significant predictor of adaptive skills (PSLE: *B* = 1.57, *p* > 0.05; Distress: *B* = -2.76, *p* < 0.05), over and above internalizing and externalizing scores as well as the other covariates. However, pregnancy stressors were not statistically significant predictors of either internalizing (PSLE: *B* = 3.11, *p* > 0.05; Distress: *B* = -0.52, *p* > 0.05) or externalizing (PSLE: *B* = -0.23, *p* > 0.05; Distress: *B* = -0.14, *p* > 0.05), above and beyond the effects of the other type of behavior problem and adaptive skills.

## Discussion

Leveraging a multisite, longitudinal cohort, the current analyses found that women’s exposure to stressful life events and their perceptions of distress during pregnancy independently predicted children’s behavior problems and adaptive functioning at age 4, demonstrating small to medium effects in the context of psychological research (Funder & Ozer, 2019). Although some studies have documented similar associations using either objective stressful events *or* subjective distress (Racine et al., 2018; Robinson et al., 2011), this study builds upon prior work and advances theories of childhood disorders to account for both types of women’s pregnancy stress in a large sample to evaluate the associations with multiple child mental health outcomes in young children. Further, these findings were significant after adjustment for a broad range of potential pre- and postnatal confounding variables, including reports of maternal perceived stress and depression at age 4, which are known to influence child functioning (Roubinov et al., 2019), increasing confidence in the associations between prenatal stress exposure and children’s outcomes found here. These findings are notable given the relatively large, multisite cohort that assessed both behavioral health problems and adaptive skills to highlight the effects of multiple types of stress on both negative and positive mental health domains.

Interestingly, women’s exposure to objectively stressful events and their subjective distress had some differential prediction of child outcomes. Both exposure to stressful events and distress independently predicted greater child Internalizing, suggesting both contribute to children’s symptoms such as anxiety and depression. However, Externalizing and Adaptive Skills appeared to be uniquely predicted by stressor type. This differential prediction may reflect differences in what each stress measure is capturing (Christensen et al., 2019). The stressful events count reflects how many unique types of major events the woman has been exposed to during pregnancy (e.g., relationship problems, housing difficulties), encompassing a range of experiences that may vary in severity, duration, and frequency. A woman may or may not experience these events as particularly stressful, depending upon her prior life experiences and coping abilities; they are however, likely to affect her available supports and resources. For example, losing a partner through divorce or death likely leads to financial and emotional strain, which reduces resources and may result in poorer nutrition, less social support, and an increase in circulating stress hormones, which can affect fetal neurodevelopment (Monk et al., 2019). Such broad reaching impacts on resources, support, and physiology may also have wider reaching and long-lasting influences on neurodevelopmental milestones implicated in psychopathology outcomes generally. On the other hand, the measure of perceived distress during pregnancy was a cross-domain (i.e., depression, anxiety, self-efficacy) composite of emotional distress the woman was consciously experiencing. Such distress may be more chronic in nature or fluctuate over time (Glynn et al., 2018), but is likely to influence her stress physiology in a manner that may affect fetal development (Monk et al., 2019). It may be that perceptions of broad ranging emotional distress specifically influence the child’s development in a manner resulting in deficits in similar domains (i.e., Internalizing Problems and Adaptive Skills). These findings provide early evidence that stressor type may be differentially related to disorder development in childhood, however, replication is needed to ascertain whether these are stable, distinct patterns.

Advancing beyond the primary focus of examining main effects, this study also explored the possibility that types of prenatal stress might interact to amplify risk for offspring. Despite a previous study finding pregnancy stressful life events were only associated with infant stress- regulation outcomes when mothers also reported higher levels of perceived stress (Bush et al., 2017), we did not find evidence for such interactions in our study. This lack of significance is notable given our relatively large sample size and may be associated with characteristics of our sample. Our sample includes a greater proportion of White, middle-to-upper income families, whereas Bush et al. (2017) found interactive effects in a sample of primarily low-income, racial-ethnic minority individuals reporting high levels of stressful experiences in their day-to-day lives (e.g., economic strain, victimization). It is possible that the interaction between exposures and perceptions during pregnancy may be detectable at high levels of environmental stress. In addition, the Bush et al. (2017) study utilized a specific measure of perceived stress, whereas the current study utilized a distress composite that included multiple forms of emotional challenges, which may operate differently than a measure specific to global stress. Future studies may benefit from isolating specific domains of perceived experience rather than using a multi-domain composite of distress.

Our exploration of whether child biological sex modified the intergenerational effects of stress advanced the limited empirical examinations of these associations to date. Theories about sex-specific mechanisms, built upon compelling animal studies, suggest that sex may be an important biological variable that signals differences in fetal brain development and placental functioning (Monk et al., 2019). Yet, only one of the sex interactions tested was significant, and that finding suggested that girls were more susceptible to the deleterious effects of maternal stressful life events during pregnancy on their preschool-aged Externalizing Problems. Although existing literature is mixed (de Bruijn et al., 2009; Hentges et al., 2019), our findings align with a few previous examinations that found exposure to psychobiological stress during pregnancy was predictive of more neurodevelopmental vulnerability in female infants (Monk et al., 2019; Sandman et al., 2013). However, given our analyses of sex interactions were for a secondary, exploratory aim, results should be interpreted with caution. We suggest additional research is required to further interpret these effects and elucidate whether potential sex-specific differences may operate through prenatal programming or postnatal socialization mechanisms.

### Strengths & Limitations

The current study has many notable strengths including evaluating associations within a large, multi-site sample with greater sociodemographic diversity than is typical in this research domain, the inclusion of multiple types of prenatal stress, the assessment of both behavioral problems and adaptive functioning, and consideration of effect modification by child sex. However, the current study also had several limitations. First, although recent evaluations suggest that retrospective report of exposure to significant life events (such as the experiences of severe illness, death of a close relative, or relationship changes) provides a valid measure of these exposures (Ramos et al., 2020), it is possible that postnatal experiences could impact the way participants remember events from their pregnancy. In the current study, we asked women about their experiences of these events during pregnancy at their child’s age 6 assessment, whereas the measure of distress was measured during pregnancy. Therefore, it is possible that the retrospective reports of events may have diminished validity, and the incongruent timing of the measurement of pregnancy SLE and distress may have affected our ability to evaluate additive effects of different types of stress. However, our study effects were similar to studies measuring both types of stress in closer proximity (Bush et al., 2017) and aligns with theoretical suppositions that types of stress are, at best, loosely related (Epel et al., 2018; Mauss et al., 2005). Second, families of color or those with strained socioeconomic resources are disproportionately exposed to a variety of stressors, and therefore, the present findings may not capture effects that would be expected in samples with greater diversity of race/ethnicity or income (Burns et al., 2015; Graham et al., 2021; Stockman et al., 2015). Third, all measures in the study were reported on by mothers, and it is possible that there may have been bias in mother’s report of their children’s problems. Notably, recent evaluations suggest a very modest effect of maternal perceptions in terms of biasing her report of child outcomes (Olino et al., 2021), however, to increase confidence, our final statistical model adjusted for current maternal depression and perceived stress to account for potential mood-related reporting bias. Shared method variance from these maternal reported measures may have potentially inflated the associations here, though psychometricians debate whether this issue may have a meaningful influence on results (Richardson et al., 2009). We also conducted sensitivity analyses to account for shared variance among the outcomes. Whereas the pregnancy stressors remained statistically significant predictors of adaptive skills, they were unrelated to internalizing and externalizing after including the other outcomes along with covariates as predictors in the models, suggesting that associations with internalizing and externalizing in our 4-year-old sample may reflect stress predictions of a more general factor underlying problem behaviors. Thus, identifying predictive associations for specific subdomains of psychopathology may be difficult to disentangle at this young age. Finally, although findings are inconsistent across samples, emerging evidence suggests exposure to environmental chemicals may contribute to children’s behavior (e.g., Day et al., 2021), and potential endocrine disrupting confounders were not adjusted for in the present analysis.

### Conclusion

The present findings highlight the importance of incorporating multiple types of pregnant women’s stress when evaluating intergenerational transmission of maternal stress on children’s mental health outcomes, as both exposure to stressful events and perceptions of distress predicted outcomes, in distinct patterns, and sometimes additively. The current work adds to an emerging literature by advancing understanding of how experiences in utero, and in early in life, can position children on a path of increased risk for psychopathology. Such work illuminates etiology and highlights potential points for early intervention, by tracing early risk for transdiagnostic mental health problems to experiences and perceptions during the prenatal period, where family-focused pre and perinatal interventions may be particularly effective in combating rising rates of child psychopathology and promote positive development.

## Supplementary Information

Below is the link to the electronic supplementary material.

Supplementary file1 (DOCX 17 KB)
