# Supplementary material for: Associations Between Maternal Stressful Life Events and Perceived Distress during Pregnancy and Child Mental Health at Age 4
Source: Res Child Adolesc Psychopathol. 2022 Mar 8;50(8):977–86. doi: 10.1007/s10802-022-00911-7 (PMC9395496; doi:10.1007/s10802-022-00911-7)
Supplement: Supplementary file 1 — Supplementary file1 (DOCX 17 KB) [file 10802_2022_911_MOESM1_ESM.docx]

**Supplemental Table 1**. Regression model results for tests of two-way interactions between PSLE, perceived distress, and child sex on

|  | | | **Externalizing** | | | | | **Internalizing** | | | **Adaptive Skills** | | |
| --- | --- | --- | --- | --- | --- | --- | --- | --- | --- | --- | --- | --- | --- |
| Effect | | | B | | | SE | | B | SE | | B | | SE |
| **Model 2** | | | | | | | |  | | |  | |  |
|  | | **Covariates** | | | |  | |  |  | |  | |  |
| Child Sex (1 = female) | | | 0.13 | | | 0.72 | | 1.56 | 0.82 | | -0.47 | | 0.79 |
| Site = UMN | | | 0.58 | | | 1.14 | | 1.98 | 1.29 | | 0.06 | | 1.26 |
| Site = UCSF | | | 0.86 | | | 1.21 | | 1.88 | 1.34 | | -0.21 | | 1.33 |
| Site = UW | | | 0.68 | | | 1.22 | | 2.12 | 1.39 | | -0.94 | | 1.35 |
| Marital Status (1 = Married/living as married) | | | 0.91 | | | 1.64 | | 1.07 | 1.86 | | -1.71 | | 1.79 |
| Gestational Age at birth (weeks) | | | -0.10 | | | 0.26 | | 0.17 | 0.29 | | 0.30 | | 0.28 |
| Gravidity | | | -0.31 | | | 0.28 | | -0.88** | 0.32 | | 0.35 | | 0.31 |
| Birthweight | | | 0.65 | | | 0.84 | | -0.51 | 0.95 | | 0.37 | | 0.92 |
| Smoking during pregnancy (1 = yes) | | | 6.02** | | | 1.87 | | 3.80 | 2.11 | | -1.68 | | 2.05 |
| Substance use during pregnancy (1 = yes) | | | 2.10 | | | 1.25 | | -0.21 | 1.42 | | 0.52 | | 1.38 |
| Mother’s Education at age 4 | | | 0.16 | | | 1.21 | | 0.83 | 1.38 | | -1.16 | | 1.34 |
| Family Income during pregnancy (in $10,000) | | | -0.01 | | | 0.01 | | 0.01 | 0.01 | | 0.03* | | 0.01 |
| Child Age at outcome (years) | | | 1.12 | | | 1.27 | | 1.70 | 1.44 | | 1.75 | | 1.40 |
| Maternal Depression at age 4 | | | 1.13 | | | 1.18 | | 1.67 | 1.33 | | 1.53 | | 1.30 |
| Maternal Perceived Stress at age 4 | | | 2.80** | | | 0.80 | | 2.01* | 0.91 | | -2.70 | | 0.89 |
|  | **Main Effects** | | |  | |  | |  |  | |  | |  |
| Pregnancy Stressful Life Events (count of types) | | | | 0.60* | | 0.26 | | .072* | 0.29 | | -0.16 | | 0.29 |
| Pregnancy Perceived Distress | | | 1.28 | | | 0.83 | | 1.99* | 0.94 | | -1.84* | | 0.91 |
|  | **Interactions** | | | | | |  |  | |  |  |  | |
| Stressful Life Events * Distress | | | | | 0.10 | | 0.42 | 0.64 | | 0.48 | -0.27 | 0.46 | |
| Stressful Life Events * Child Sex | | | | | 1.14* | | 0.49 | 0.14 | | 0.56 | -0.37 | 0.54 | |
| Distress * Child Sex | | | | | 0.51 | | 1.47 | 1.14 | | 1.67 | -1.82 | 1.67 | |
|  | | | ***R^2^ = 0.17*** | | | | | ***R^2^ = 0.14*** | | | ***R^2^ = 0.11*** | | |

children’s age 4 outcomes.
